# Eradication of *Pseudomonas aeruginosa* Biofilms by Atmospheric Pressure Non-Thermal Plasma

**DOI:** 10.1371/journal.pone.0044289

**Published:** 2012-08-31

**Authors:** Mahmoud Y. Alkawareek, Qais Th. Algwari, Garry Laverty, Sean P. Gorman, William G. Graham, Deborah O'Connell, Brendan F. Gilmore

**Affiliations:** 1 School of Pharmacy, Queen’s University of Belfast, Belfast, United Kingdom; 2 Centre for Plasma Physics, Queen's University of Belfast, Belfast, United Kingdom; 3 York Plasma Institute, Department of Physics, University of York, York, United Kingdom; 4 Department of Electronics, College of Electronic Engineering, University of Mosul, Mosul, Iraq; University of Malaya, Malaysia

## Abstract

Bacteria exist, in most environments, as complex, organised communities of sessile cells embedded within a matrix of self-produced, hydrated extracellular polymeric substances known as biofilms. Bacterial biofilms represent a ubiquitous and predominant cause of both chronic infections and infections associated with the use of indwelling medical devices such as catheters and prostheses. Such infections typically exhibit significantly enhanced tolerance to antimicrobial, biocidal and immunological challenge. This renders them difficult, sometimes impossible, to treat using conventional chemotherapeutic agents. Effective alternative approaches for prevention and eradication of biofilm associated chronic and device-associated infections are therefore urgently required. Atmospheric pressure non-thermal plasmas are gaining increasing attention as a potential approach for the eradication and control of bacterial infection and contamination. To date, however, the majority of studies have been conducted with reference to planktonic bacteria and rather less attention has been directed towards bacteria in the biofilm mode of growth. In this study, the activity of a kilohertz-driven atmospheric pressure non-thermal plasma jet, operated in a helium oxygen mixture, against *Pseudomonas aeruginosa in vitro* biofilms was evaluated. *Pseudomonas aeruginosa* biofilms exhibit marked susceptibility to exposure of the plasma jet effluent, following even relatively short (∼10′s s) exposure times. Manipulation of plasma operating conditions, for example, plasma operating frequency, had a significant effect on the bacterial inactivation rate. Survival curves exhibit a rapid decline in the number of surviving cells in the first 60 seconds followed by slower rate of cell number reduction. Excellent anti-biofilm activity of the plasma jet was also demonstrated by both confocal scanning laser microscopy and metabolism of the tetrazolium salt, XTT, a measure of bactericidal activity.

## Introduction

Microbial biofilms are organised, multicellular communities held together by a self-produced matrix forming architecturally complex structures [1]–[3]. Biofilms are ubiquitous in both natural and pathogenic ecosystems [4] and may be present on almost every biotic or abiotic surface or interface [2]. The process of biofilm formation begins with cellular attachment to a substratum, and formation of microcolonies on the surface, which become embedded within a self-produced extracellular, hydrated matrix and which subsequently differentiate into a mature biofilm, These exhibit architecturally defined, complex three-dimensional structures and a functionally heterogeneous bacterial population [4], [5]. Dispersal mechanisms facilitate colonisation of new niches [2], including repopulation of surfaces following sub-lethal antimicrobial challenges.

Biofilms are estimated to be implicated in around 80% of all chronic human infections [6] and are important mediators of healthcare-associated infections [7], around half of which are related to the use of an indwelling medical device [8]. In 2008, more than 4 million patients acquired healthcare-associated infections in European hospitals; which resulted in about 37000 deaths as a direct consequence [7]. In the US, the number of such infections was more than 1.7 million in 2002 with almost 100,000 associated deaths [9]. Infections related to medical devices were the first clinical infections to be identified as having biofilm aetiology [5], [10]. With many millions of medical devices being used each year [11], biofilms constitute a significant public health risk for patients requiring such devices [12]. Among these devices are: intravenous catheters, prosthetic heart valves, joint prostheses, peritoneal dialysis catheters, cardiac pacemakers, cerebrospinal fluid shunts, urethral catheters, urinary stents and endotracheal tubes, which all have an intrinsic risk of surface-associated infections [5]. Biofilms have also been associated with many other conditions, on biotic surfaces, including dental plaque, upper respiratory infections, peritonitis, and urogenital infections [11].

Biofilms constitute a protected mode of bacterial growth [2] and bacteria within biofilms typically exhibit significantly enhanced tolerance to antimicrobial challenge and host defences, compared to planktonic bacteria of the same species [2], [5], [13]–[15]. Indeed, bacteria in biofilms can be up to a thousand times more resistant to antimicrobial challenge than the planktonic cells of the same species [16], [17]. This recalcitrance to antimicrobial challenge can explain why biofilm-mediated infections often fail to respond to conventional antibiotic treatment regimens [14]. Many mechanisms of biofilm resistance to antimicrobial agents have been proposed; a first mechanism is the failure of antimicrobial agents to penetrate into the whole structure of the biofilm as a result of the barrier properties offered by the polymeric matrix [2], [5], [13]. Another mechanism explaining biofilm resistance is that a large number of microorganisms within a biofilm experience nutrient depletion which renders them slow-growing or metabolically inactive [2], [13]. Most antimicrobials require at least some degree of cellular activity to be effective; since their mechanism of action usually relies on disrupting different microbial metabolic processes [5]. The third mechanism, of biofilm resistance, is related to the presence of subpopulations that exhibit distinct resistant phenotypes [2], [5]. These subpopulations are frequently referred to as “persister cells” [18], [19].

*Pseudomonas aeruginosa* is an opportunistic Gram negative pathogen. The ability of this pathogen to survive in multiple niches and to utilize many naturally occurring compounds as energy sources makes it one of the most ubiquitous bacteria in both the environment and the clinical setting, where it contaminates the floor, bed rails, and sinks, and the skin of patients and healthcare personnel [20]. Unsurprisingly, in the European Prevalence of Infection in Intensive Care Study, this bacterium was found to be responsible for up to 28% of nosocomial infections in intensive care units [21]. The majority of these infections were found to affect immunocompromised patients or those having severe underlying diseases like cystic fibrosis and severe burns in addition to those who are in contact with contaminated medical devices [21]. Unfortunately, such nosocomial infections are frequently life threatening and often challenging to treat; especially with the frequent emergence of resistance to multiple drugs in this pathogen [22]. This resistance can emerge gradually during exposure to antipseudomonal antibiotics [23], this emergence was reported in 27–72% of patients with initially susceptible *P. aeruginosa* isolates and usually results in higher morbidity, mortality and economic burden [22].

As an antimicrobial strategy, atmospheric pressure non-thermal plasmas benefit from simple design, relatively low capital and operational cost [24], utilisation of non-toxic gases [25], operating at gas temperatures at or near room temperature [24]–[26], and the absence of harmful residues [24]. Importantly, these plasmas produce large quantities of microbicidal active agents [27]; which include a mixture of charged particles (positive and negative ion and electrons), chemically reactive species, UV radiation and electromagnetic fields [28]. The diversity and the small size of these active agents are believed to target multiple cellular components and metabolic processes in microorganisms and therefore make the emergence of resistance mechanisms less likely.

The exact mechanisms driving plasma-mediated bacterial inactivation are not yet well understood [29], [30]. However, several plasma products are believed to play a role in this process, these products include reactive oxygen species (ROS) [29]–[31], reactive nitrogen species (RNS) [31], ultraviolet radiation (UV) [30] and charged particles [30]. Among the ROS believed to be involved in this process are ozone, atomic oxygen, single delta oxygen, superoxide, peroxide, and hydroxyl radicals [30], [32]. Although many of the aforementioned ROS have documented antibacterial activities through their interactions with different cellular components [30], [33], [34], it is highly important to consider the additive and synergistic effects of these species with each other’s and with other plasma products like UV radiation and charged particles, in such a physically and chemically complex environment, before any successful conclusions can be drawn about the responsible cellular inactivation processes.

The use of atmospheric pressure non-thermal plasmas has been evaluated for a number of biomedical applications, including: wound healing [26], [35], blood coagulation [35], [36], skin regeneration [35], tooth bleaching [36], and apoptosis of cancer cells [35], [36]. In addition, many atmospheric pressure plasma systems have been studied and proven to be effective in terms of microbial inactivation and even surface decontamination and sterilization [24], [26], [35]–[38]. However, most of these studies have been carried out using planktonic bacteria, which do not represent the entire spectrum of bacterial growth and survival in the natural as well as clinical settings. Whilst a number of studies have examined the effect of atmospheric pressure non-thermal plasmas on microbial biofilms [39]–[47], this study presents a comprehensive investigation of the activity of an in-house designed kHz-driven atmospheric pressure non-thermal plasma jet for the *in vitro* eradication of the clinically significant *P. aeruginosa* biofilms grown on inanimate surfaces. Multiple approaches were adopted to evaluate the bacterial cell viability prior to and after plasma treatment. These approaches are based on colony count method, XTT assay and LIVE/DEAD differential staining followed by confocal laser scanning microscopy. Furthermore, the effect of changing the plasma operating frequency on the anti-biofilm activity of the plasma jet was also investigated. Although the exact explanation for the difference in plasma activity upon changing the frequency was not provided in this manuscript since it needs further investigations, to our knowledge, this is the first research paper to date to report the effect of changing the frequency, within the same jet configuration, on biofilm eradication by plasma; which adds useful information about tailoring this approach to suit different biofilm-related applications.

## Materials and Methods

### Bacterial Strain(s) & Growth Conditions

*Pseudomonas aeruginosa* PA01 (ATCC BAA-47, obtained from the American Type Culture Collection) was stored at −70°C in Microbank vials (Pro-Lab Diagnostics, Cheshire, UK) and was subcultured in Müller-Hinton Broth (MHB) several times prior to conducting the microbiological assessments.

### Plasma Source

**Figure 1:**
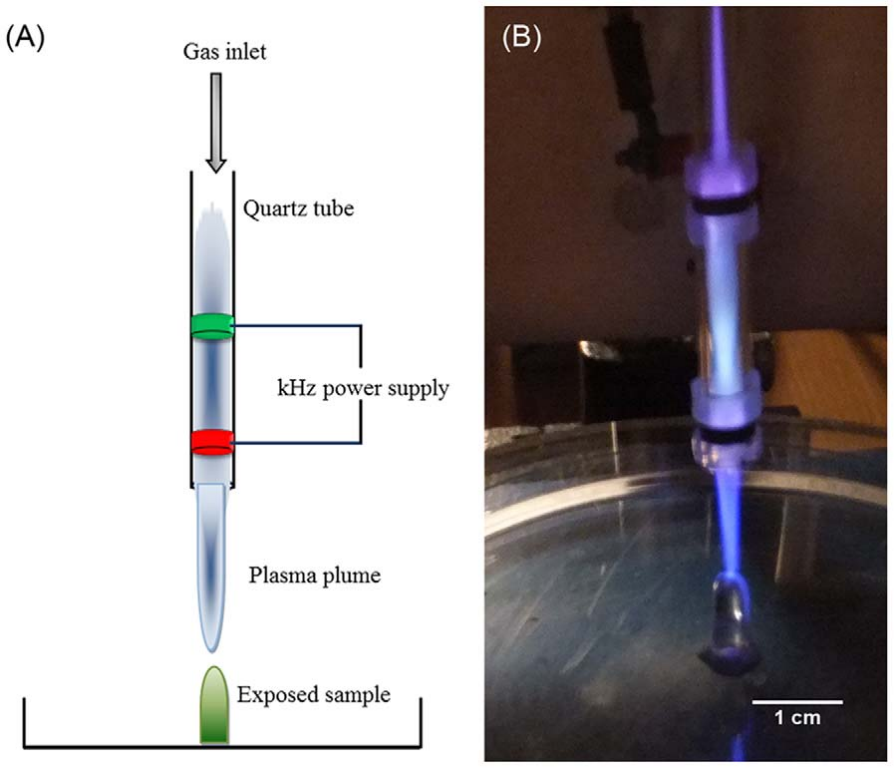
The plasma jet used in this study. (A) Schematic diagram of the plasma jet. (B) Photograph of the plasma jet interacting with a biofilm sample.

A schematic diagram and a photograph of the plasma jet used in this study, as previously described in [43], [48], are presented in Figure 1. It consists of a quartz tube with inner and outer diameters of 4 mm and 6 mm, respectively. Two copper electrodes (2 mm wide) encircle the tube, separated by 25 mm. For the experiments presented here the output of a high voltage pulse source (Haiden PHK-2k), operating at variable repetition frequencies, of between 20 and 40 kHz, and voltage amplitude 6 kV, is applied to the downstream electrode, which is 5 mm from the end of the plasma tube. The upstream electrode is grounded. The plasma jet was operated with a gas mixture of 0.5% oxygen and 99.5% helium, at a total flow rate of 2 Standard Litres per Minute (SLM) into ambient air. This plasma jet can be observed to generate an intense core plasma between the two electrodes and a luminous plume, which under the operating conditions discussed here, extends up to several centimetres beyond the exit of the tube. Spatially and temporally resolved images in the main plasma production region and plume regions confirm the presence of streamer-like behaviour ([48]–[52], which is in good agreement with the model proposed by Lu and Laroussi [49].

### Inhibition Zone Determination

Inhibitory zones formed on *P. aeruginosa* lawns on solid agar cultures following exposure to the plasma plume were determined as described previously [43]. After exposure to the plasma, Mueller Hintonagar (MHA) plates which had been streaked with a phosphate buffered saline-diluted overnight culture of *P. aeruginosa* were incubated at 37°C for 24 hours in a static incubator and the diameter of zones of inhibition measured using Vernier callipers.

### Biofilm Growth

For survival curves construction and XTT assay experiments, bacterial biofilms were grown on the peg lid of Calgary Biofilm Device (commercially available as the MBEC Assay™ for Physiology & Genetics (P & G), Innovotech Inc., Edmonton, Alberta, Canada). An overnight culture of PA01 was adjusted to an optical density (OD_550_) equivalent to 1×10^7^ cfu/ml. The standardized bacterial suspension was used to inoculate the Calgary Biofilm Device (with 150 µl in each well) which was then incubated at 37°C for 48 hours in a humidified compartment within an orbital incubator. The bacterial inoculum was replaced by fresh growth medium (i.e. MHB) after the first 24 hours of incubation. At the end of 48 hours of incubation, individual pegs were broken off the lid with sterile pliers and gently rinsed with 200 µl of PBS for 1 minute to get remove of any planktonic or loosely adhered bacteria before exposure to the plasma jet.

For microscopic examination, bacterial biofilms were grown on polycarbonate coupons (10 mm diameter) in a continuous flow cell chamber (FC 271-AL-3×10 Dual Channel Coupon Evaluation Flow Cell, BioSurface Technologies Corp., Bozeman, Montana, USA) for 3 days. Initially, fresh growth Mueller Hinton broth (MHB) was allowed to perfuse the flow cell at a rate of 0.1 ml/minute for 24 hours, after which time the perfusion was arrested and 1 ml of a mid-logarithmic phase bacterial suspension was injected into the flow cell chamber and left for 1 hour (under static conditions) to allow for adherence of bacterial cells onto the coupon surface. Following cell adherence, the flow cell chamber was perfused with fresh MHB at a rate of 0.1 ml/minute for 3 days. At the end of the biofilm growth period, a final rinsing step was carried out by running 0.9% NaCl solution through the flow cell at a rate of 1.0 ml/minute for 10 minutes before removing the coupons bearing mature biofilm, using sterile surgical tweezers, and exposure to the plasma treatment.

### Treatment Conditions

Individual pegs, bearing 48 hr PA01 biofilms, were exposed to the plume of the plasma jet for varying periods of time (0, 15, 30, 45, 60, 120, and 240 seconds) as described previously [43]. The treatment was repeated in triplicate at each time point. The distance between the end of the plasma tube and top of the peg was fixed to 15 mm. The atmospheric pressure plasma jet was operated at a frequency of either 20 or 40 kHz at a set voltage of 6 kV. For determining bacterial inhibition zones resulting from plasma exposure, the bacteria seeded agar plates were placed under the plasma plume at a distance of 25 mm away from the end of the plasma tube for the following time intervals: 0, 120, and 240 seconds. After plasma exposure, the plates were incubated and photographed as mentioned previously. Mature biofilms grown on polycarbonate coupons in the flow chamber described above were exposed to the 20 kHz plasma jet under the same operating conditions but at two time points (60 seconds and 240 seconds). In addition, a gas-only exposure (with the power supply switched off) was carried out as a control.

### Cell Viability Determination

After plasma exposure, the pegs were replaced in the wells of a 96-well Microtiter plate containing 200 µl PBS in each well and sonicated [17] for 10 minutes in order to dislodge the biofilm cells from the pegs and to re-suspend surviving bacteria in PBS to permit viable counting. Following sonication, the pegs were discarded and the resultant bacterial suspensions were used to determine the viability of surviving bacterial cells.

After bacterial biofilm exposure to the plasma jet, the viability of surviving cells was quantitatively determined using two methods: standard colony count method and XTT viability assay. In the standard colony count method, the recovered bacterial suspensions were 10-fold serially diluted in a 96-well microtiter plate using sterile PBS, and three aliquots (20 µl each) from each well were spotted on the surface of MHA plate. After incubating the MHA plates at 37°C for 24 hours, the number of colonies originated from each spot was counted using a colony counter. The number of surviving cells was calculated as colony forming unit per peg (cfu/peg) and survival curves were constructed based on these values. Furthermore, percentage cell killing was calculated by comparing the number of surviving cells in each sample with the number of bacterial cells present in the untreated samples (zero exposure time) prepared under the same conditions.

XTT viability assay (XTT based in vitro Toxicology Assay Kit, Sigma-Aldrich Company Ltd., Dorset, UK) was carried out as follows: an XTT stock solution was prepared by reconstituting a kit vial (containing 5 mg of XTT with 1% PMS) with 5 ml of PBS. 50 µl aliquots of the recovered bacterial suspensions (after plasma exposure) were transferred into the wells of a 96-well microtiter plate containing 50 µl of growth media (MHB), and then 20 µl of the XTT stock solution was added to each well. After incubating the microtiter plate at 37°C for 5 hours in an orbital incubator, the absorbance at 450 nm was measured, against blank controls (containing 50 µl PBS, 50 µl MHB, and 20 µl XTT solution), using a plate reader (BioTek EL808 Microplate Reader, BioTek UK, Bedfordshire, UK) in order to quantify XTT metabolic product, the intensity of which is proportional to the number of respiring cells. The percentage cell killing at each time point was calculated by comparing the absorbance of the samples representing that time point with the absorbance representing the untreated control samples.

### Confocal Scanning Laser Microscopy

Following exposure of the biofilms grown on polycarbonate coupons to the plasma jet, biofilms were stained with LIVE/DEAD BacLightBacterial Viability Kit (Molecular Probes, Eugene, OR, USA), and examined by confocal laser scanning microscope (Leica TCS SP2 Confocal Microscope, Leica Microsystems, UK). Z-stacks of confocal images were rendered into 3D mode using Volocity software (PerkinElmer, UK).

## Results and Discussion

### Bacterial Growth Inhibition Zones

**Figure 2:**
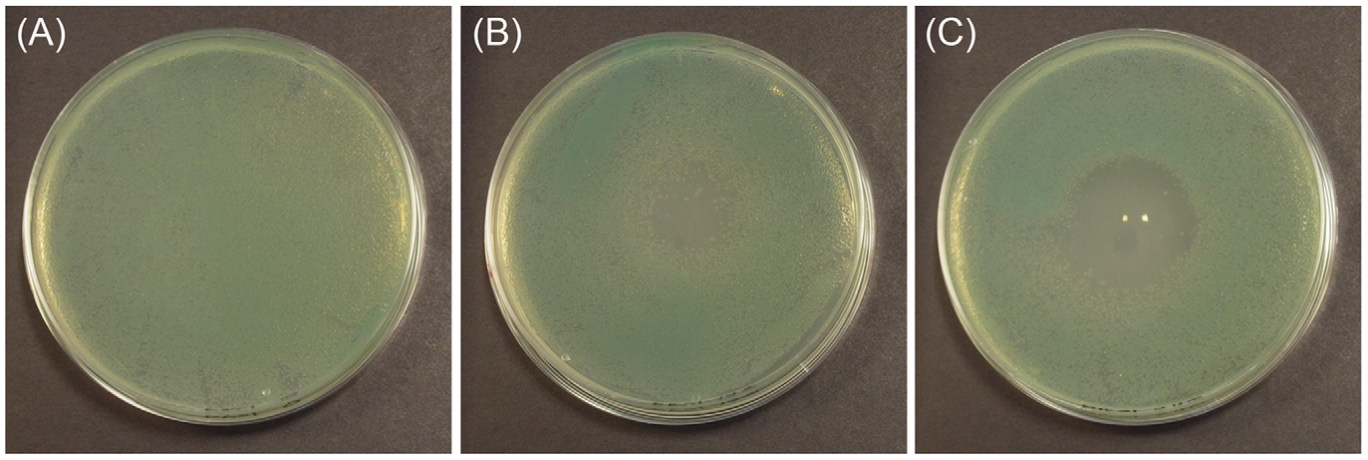
Bacterial growth inhibition zones. *P. aeruginosa* cell suspensions were spread over MHA plates (9 cm in diameter). The seeded plates were exposed to the 20 kHz plasma jet for (A) 0 s, (B) 120 s, and (C) 240 s and then incubated at 37°C for 24 hours. After incubation, photographs of agar plates, showing bacterial growth inhibition zones, were taken using a digital camera.

In order to visually demonstrate the effect of the investigated atmospheric pressure plasma jet on the viability of bacterial cells, MH agar plates seeded with *P. aeruginosa* were exposed to the plasma plume and incubated overnight. As shown in Figure 2, the plasma-exposed plates showed significant bacterial inhibition zones which indicate the extent of bactericidal activity of the plasma jet, against planktonic *P. aeruginosa* spread over the surface of the agar.

Although the maximum diameter of the plasma plume observed in the current jet is less than 5 mm, inhibition zones of several centimetres are obtained; which indicates that plasma-derived active species are not confined within the visible plasma plume, rather they are present in a larger volume around it. The observable inhibition zone diameter increased with increasing exposure time. This is most likely due to decreasing densities of the active bactericidal species as a function of distance from the centre of the visible plasma plume, as these species interact with components of ambient air. Therefore areas at the periphery of the inhibition zone would be expected to require longer exposure times in order to inactivate the bacteria present.

### Biofilm Eradication by 20 kHz Plasma and Its Survival Curve

**Figure 3:**
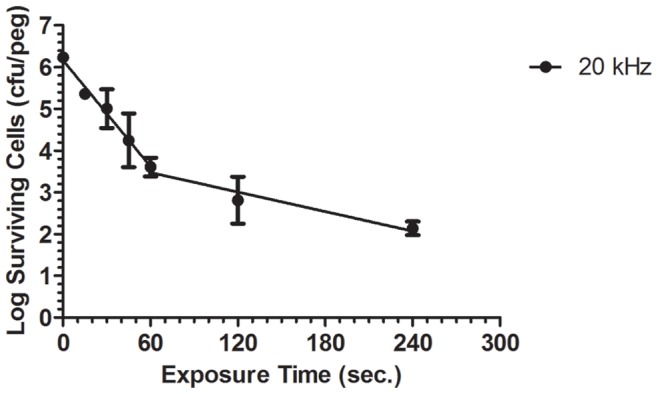
Survival curve of biofilm treated with 20 kHz plasma. 48-hour *P. aeruginosa* biofilms, grown on Calgary Biofilm Device, were exposed to the 20 kHz plasma jet for up to 4 minutes. The number of biofilm surviving cells in each sample was then calculated using colony count method and used to construct the log survival curve. (Each point represents the mean of 3 values ± SE).

In biofilm eradication studies, the plasma jet under investigation, operated at 20 kHz and 6 kV produced more than 4-log (99.99%) reduction in the number of viable cells of *P. aeruginosa* biofilm within 4 minutes of exposure, as shown in Figure 3. In an earlier short communication, we observed complete eradication of *P. aeruginosa* biofilms after 10 minutes of exposure using the same plasma jet operating at 20 kHz and 6 kV [43]. These results are superior to those reported for other atmospheric pressure plasma jets against various Gram negative bacterial biofilms; where less than a 2-log (99%) reduction was achieved in 4 minutes with *Chromobacterium violaceum* [39] and *Neisseria gonorrhoeae* [53] biofilms. In another study, only 40 fold reduction in the number of *E. coli* biofilm cells was achieved after 40 minutes of exposure to another setup of atmospheric pressure plasma [41], even though *E. coli* biofilms had been reported to be more susceptible, toward the plasma jet investigated in this study, than *P. aeruginosa* biofilms [43]. Treatment of fungal biofilms using three different plasma systems was also reported in [46]; where the most efficient system showed 5-log reduction after 10 minutes of plasma exposure, but complete biofilm eradication was not achieved with any of the three plasma systems [46].

On the other hand, activity of a radiofrequency plasma needle was evaluated against *Streptococcus mutans* biofilms [44]. Although this plasma needle was shown to have a good activity against biofilms, grown under certain conditions, no enumeration of surviving biofilm cells was carried out in this study. In another study, microwave-induced argon plasma has been shown to completely eradicate *E. coli*, *S. epidermidis* and MRSA biofilms after 20 seconds of exposure [45], but quantitative analysis of biofilm surviving cells was based on crystal violet (CV) assay. While CV assay is a good indicator of the total attached biomass, it is poorly suited to evaluate killing of biofilm cells [54], [55]. In a recent study, oral biofilms formed *in situ* on titanium discs were removed by microwave-driven nonthermal plasma, however, “complete removal” was only achieved after another treatment with air/water spray followed by a second cycle of plasma treatment [47]. Although these three studies present valuable information on the use of atmospheric pressure plasma for biofilm eradication, no survival curves or log-reduction values were reported which makes it hard to compare the efficiencies of these plasma systems with that of the system being investigated in the current study.

The survival curve presented in Figure 3 is characterised by a biphasic trend in which there are two bacterial inactivation phases; each with a distinct decimal reduction time (D-value, the time taken to reduce the bacterial population by 90% or a one log reduction). The first phase occurring during the initial 60 seconds of plasma exposure is characterised by a rapid reduction in the number of surviving cells with a D-value of 23.57 seconds. After 60 seconds of exposure, a second inactivation phase starts with a lower rate of bacterial destruction reflected by a higher D-value of 128.20 seconds. These data concur with similar biphasic survival curves reported for *Chromobacterium violaceum* [39] and *Neisseria gonorrhoeae* [53] biofilms, on exposure to different setups of atmospheric pressure plasma. The slower rate of biofilm reduction observed after 60 seconds of plasma exposure may result from the protection provided by the polymeric matrix surrounding cells in deep layers of the biofilm, or may be caused by the shielding effect of cellular debris produced from the plasma-lysed cells at the surface of biofilms, as previously proposed [39].

### Effect of Plasma Frequency Variation on Its Anti-biofilm Activity

**Figure 4:**
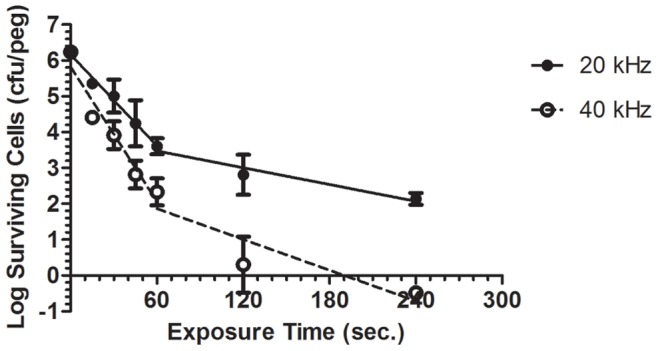
Survival curves of biofilms treated with 20 kHz vs. 40 kHz plasma. Comparison between log survival curves of *P. aeruginosa* biofilm cells, constructed as described above, upon exposure to both 20 kHz and 40 kHz plasma jet. (Each point represents the mean of 3 values ± SE).

As discussed above the kHz-driven plasma jet used in this study produces a pulsed plasma jet or ‘plasma bullets’ rather than a continuous plasma plume [48], [49], [56]. Thus a higher frequency results in more plasma pulses and effective plasma on-time; hence on time average a higher density of the plasma reactive species will be delivered [57]. It would therefore be expected that increasing the frequency (in this case, by a factor of 2, from 20 kHz to 40 kHz) would yield improved plasma-mediated biofilm eradication by increasing the effective plasma ‘dose’ delivered. Figure 4 demonstrates that 40 kHz plasma resulted in a significantly enhanced inactivation rate of *P. aeruginosa* biofilms, with complete biofilm eradication after only 4 minutes of plasma exposure. This would indicate that biofilm inactivation by atmospheric pressure plasma is ‘dose’ dependent, besides being time dependent.

**Table 1:** D-values of 20 kHz and 40 kHz plasma jets against *P. aeruginosa* biofilm cells.

	D-Value (sec)	
Operating Frequency	Phase 1	Phase 2
20 kHz	23.57	128.20
40 kHz	15.97	69.79

Through varying the plasma pulse repetition rate, at a constant applied voltage, we can increase the dissipated power into the plasma, while E/N remains unchanged. However, it is also important to consider the recombination chemistry of the plasma afterglow since the biofilm is exposed to the time averaged plasma. Species have varying lifetimes and some species densities will increase, while others will dramatically decrease in the plasma-off phase. Metastable molecular singlet delta oxygen (SDO) is an important ROS and known agent in numerous biochemical processes, it also has an extremely long radiative lifetime of more than 75 minutes in the gas phase. The absolute density of SDO, as measured through infra-red emission spectroscopy in the same plasma source, while in general increases with increasing frequency, between 20 kHz and 40 kHz remains constant at 2×10^14^ cm-3 [32]. Since the measured gas temperature in the plasma increases with pulse repetition rate we might expect a reduction in ozone density; the dependence of ozone density on gas temperature is well known. With increasing gas temperature, the ozone production rate decreases and its destruction rate increases, both leading to a lower ozone density [58]. From this we can conclude that the mechanisms responsible for enhanced biofilm inactivation with pulse repetition rate are complex and it probably not solely an individual species responsible.

The survival curve for the 40 kHz plasma also exhibits a biphasic behaviour similar to the 20 kHz plasma but with lower D-values of 15.97 seconds and 69.79 seconds for the first and the second inactivation phases, respectively, compared to 23.57 and 128.20 seconds for 20 kHz (Table 1). A noteworthy point related to the D-values is that doubling the operating frequency from 20 kHz to 40 kHz resulted in reducing the D-value of the second phase by almost half.

Unfortunately, doubling the plasma operating frequency increased not only the dose of active species delivered by the plasma but also the gas temperature of plasma discharge. While the 20 kHz plasma plume exhibited a gas temperature, measured as described by [59], around 39°C, at 40 kHz the gas temperature of the plasma jet was about 57°C. Although measuring the “local biofilm temperature” would be an interesting way of studying its effect in the eradication process, the determination of this temperature is not practically feasible because of the micro nature of bacterial biofilms. Nevertheless, since the biofilm is exposed to the plasma in an open ambient environment and under a continuous plasma gas flow, it is less likely that local temperature of the exposed biofilm will significantly exceed that of the plasma gas temperature. However, the higher temperature, observed at 40 kHz, may limit potential applications of this discharge when operating at such higher frequencies, especially for heat sensitive materials and viable tissues, but further modifications of this discharge setup, that may reduce its gas temperature, could overcome this issue.

**Figure 5:**
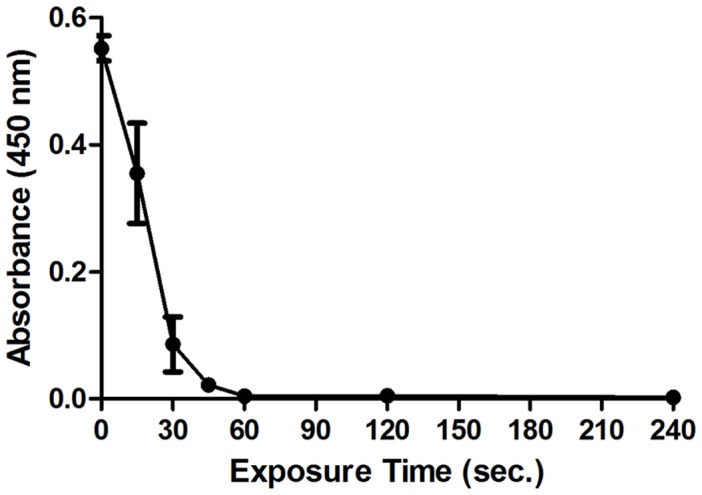
Absorbance of XTT-assay product. 48-hour *P. aeruginosa* biofilms, grown on Calgary Biofilm Device, were exposed to the 20 kHz plasma jet for up to 4 minutes. After plasma exposure, bacterial cells were dislodged off the pegs into PBS buffer by sonication. 50 µl aliquots of the recovered bacterial suspensions were then mixed with 50 µl of MHB and 20 µl of XTT stock solution and incubated at 37°C for 5 hours. After incubation, the absorbance at 450 nm was measured to quantify XTT metabolic product, the intensity of which is proportional to the number of viable (respiring) cells. (Each point represents the mean of 3 values ± SE).

**Figure 6:**
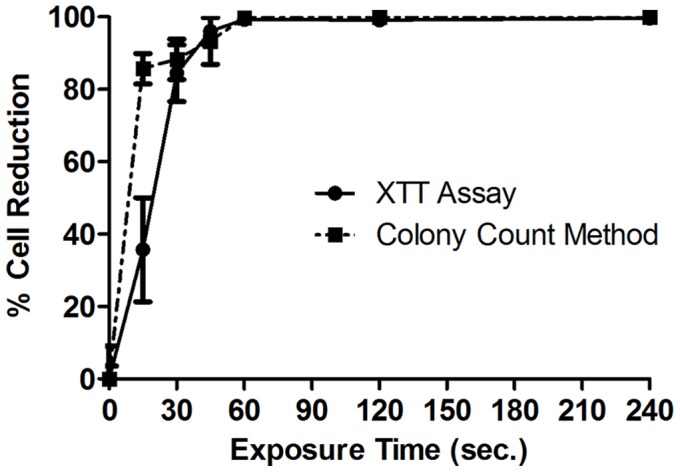
Percentage cell reduction curves based on colony count method vs. XTT assay. Percentage cell reduction curves of *P. *aeruginosa biofilm cells upon exposure to the 20 kHz plasma jet. The dotted line is based on the standard colony count method whereas the solid line is based on the XTT assay. (Each point represents the mean of 3 values ± SE).

**Figure 7:**
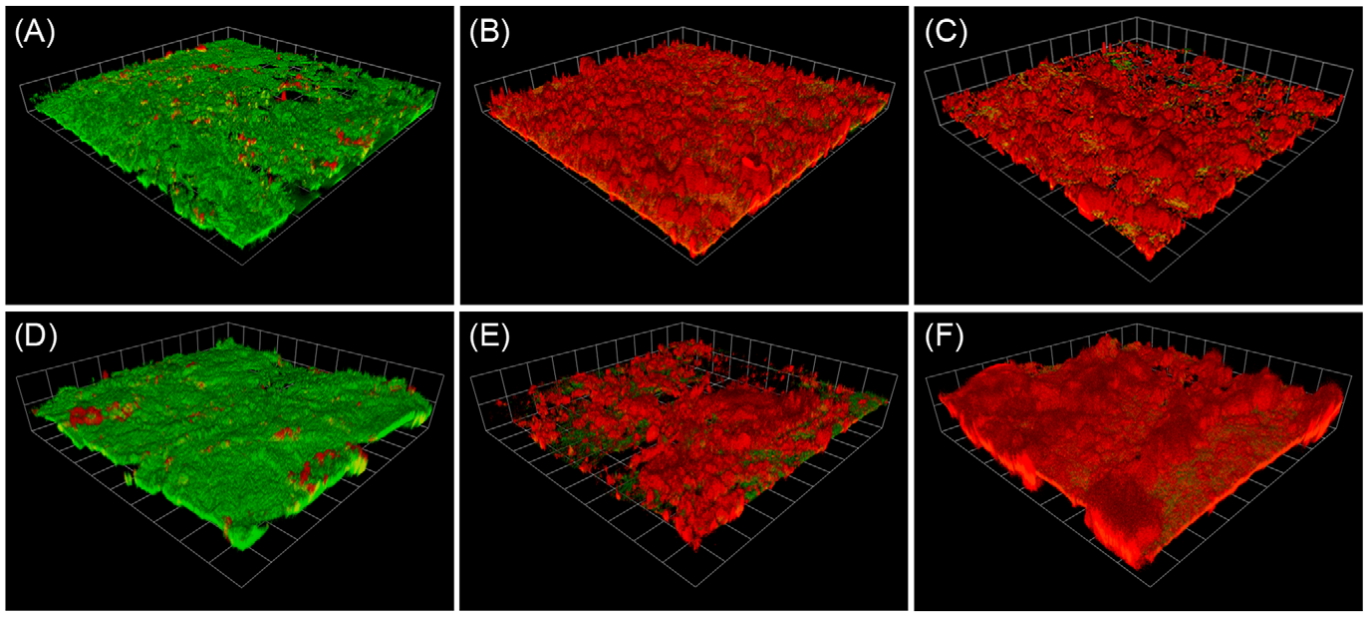
CLSM images of the plasma treated biofilms. 3D rendered confocal laser scanning micrographs of 3-day *P. aeruginosa* biofilms, grown on polycarbonate coupons, exposed to the 20 kHz plasma jet for 0s (A and D), 60 s (B and E), and 240 s (C and F). Green colour indicates surviving cells whereas red colour indicates dead cells. Magnification power is 200x (a-c) and 600x (d–f).

### Cell Viability Determination by XTT Assay

The XTT assay has been frequently used for the quantification of bacteria and bacterial biofilms [42], [60] where it detects the presence of cells that are viable and metabolically active [42] regardless of being culturable. The viability of *P. aeruginosa* biofilms treated with a 20 kHz plasma discharge was evaluated using this method. After the samples had been prepared, as previously described, absorbance at 450 nm was measured to quantify the presence of orange-coloured formazan product which results from XTT reduction by metabolically active cells, to estimate the number of surviving cells. Absorbance values (at 450 nm) measured after exposing *P. aeruginosa* biofilms to the plasma jet for different time intervals are presented in Figure 5. The values continuously drop with increasing the time of plasma exposure to reach almost nil after 60 seconds which indicates the continuous reduction in the number of surviving cells upon plasma exposure. This supports the evidence for the inactivation effect exhibited by the plasma against the bacterial biofilm. Percentage cell reduction values calculated based on XTT assay show good correlation with those obtained by the colony count method (Figure 6). One major difference between results of the two methods lies in the 15-seconds exposure point; the colony count method indicates that 85% cell killing was achieved at this point, while the value was about 36% from the XTT assay results. This finding may indicate that plasma exposure for this period of time provides a sub-lethal dose which may render some of the biofilm cells non-culturable but still viable, which was also referred to in [39].

### Live/Dead Staining and Confocal Microscopy Examination

In addition, *P. aeruginosa* biofilms were stained with Live/Dead BacLight bacterial viability staining kit for examination by confocal laser scanning microscope. The viability kit consists of two dyes; green-fluorescent SYTO 9 which stains live bacteria, and red-fluorescent propidium iodide which only penetrates non-viable bacterial cells with damaged/perturbed cell membranes. The 3D rendered confocal images of the plasma exposed biofilms, presented in Figure 7 further supports the findings of the other experiments, namely the biofilm viable counting and XTT assays. While unexposed biofilm has been shown to be predominantly green (viable), the fraction of red colour (non-viable) increased upon increasing plasma exposure time such that the 240 s plasma exposed biofilm was almost entirely stained red; indicating that vast majority of biofilm cells were by then rendered non-viable. On the other hand, the maximum biofilm thickness observed in the biofilm areas examined by the confocal microscope was between 40 µm and 80 µm. After 240 s plasma exposure, cells in the whole biofilm thickness were affected which indicates the ability of the active plasma species to penetrate deeply into the biofilm and inactivate the cells within.

### Conclusions

In this study a kHz-driven cold atmospheric pressure plasma jet has proven to be highly efficient in the *in vitro* inactivation of *P. aeruginosa* biofilms. A greater than 4-log (99.99%) reduction in the number of viable cells was achieved within 4 minutes of exposure to the plasma jet when operated at 20 kHz. While increasing the operation frequency to 40 kHz resulted in a complete eradication of the bacterial biofilm at 4 minutes. Survival curves, under both operating frequencies, exhibit a biphasic trend with a rapid decline in the number of surviving cells in the first 60 seconds followed by a slower rate of bacterial cell inactivation. Percentage cell reduction values obtained by XTT assay were, generally, in agreement with those obtained by colony count method with an exception at the early stage of exposure, this difference might suggest the presence of complex changes in cell physiology prior to their complete destruction. Confocal microscopy examination, preceded by a differential staining with LIVE/DEAD BacLight Bacterial Viability Kit, has supported the evidence of the bacterial inactivation effect exerted by the plasma jet through the whole thickness of *P. aeruginosa* biofilms. Plasma reactive species are highly reactive and relatively short-lived; although they have proven to exert an activity through full thickness of the bacterial biofilm, they are not expected to penetrate a host tissue or a thick substance. Therefore, the plasma application suggested by this study lies within the area of surface decontamination/sterilisation and not intended to cover biofilms protected by thick living tissues or growing in body cavities.

On the other hand, the comparison between plasma-induced effects at the two frequencies is difficult since the gas temperature in the plasma plume increased with frequency. More detailed measurements are required in order to quantify specific plasma species for an improved understanding of the plasma environment. Although a comprehensive and systematic understanding of the mechanisms underlying biofilm eradication and bactericidal activity by atmospheric pressure non-thermal plasmas will prove necessary for the translation of this technology to clinical applications, atmospheric pressure non-thermal plasmas would appear to offer a promising, novel and highly efficient strategy for the control of microbial biofilms on inanimate surfaces, such as biomaterials employed in the manufacture of indwelling medical devices, as well as viable tissues.
